# From fungal diversity to antimicrobial innovation: the potential of biotransformation in the era of resistance

**DOI:** 10.3389/fmicb.2026.1847633

**Published:** 2026-06-10

**Authors:** Juan Diego Ribeiro de Almeida, Rita de Cassia Santos da Silva, Vanessa da Silva Bindá, Izabela de Mesquita Bárcia Moreira, Carlos Daniel Santos de Sousa, Robert Langlady Lira Rosas Filho, João Paulo Alves Silva, Hagen Frickmann, Érica Simplício de Souza, João Vicente Braga de Souza

**Affiliations:** 1 Multicenter Graduate Program in Biochemistry and Molecular Biology, Amazonas State University, Manaus, Brazil; 2 Medical Mycology Laboratory, National Institute for Amazonian Research, Manaus, Brazil; 3 Department of Chemical Engineering, Lorena School of Engineering, University of São Paulo, Lorena, São Paulo, Brazil; 4 Institute for Medical Microbiology, Virology and Hygiene, University Medicine Rostock, Rostock, Germany; 5 Department of Microbiology and Hospital Hygiene, Bundeswehr Hospital Hamburg, Hamburg, Germany; 6 School of Technology, Amazonas State University, Manaus, Brazil

**Keywords:** antimicrobial resistance, biocatalysis, biotransformation, filamentous fungi, natural products, peroxygenases

## Abstract

Antimicrobial resistance (AMR) continues to outpace the development of new anti-infective agents, particularly against priority bacterial pathogens such as *Acinetobacter baumannii, Pseudomonas aeruginosa, Klebsiella pneumoniae*, and *Staphylococcus aureus*, as well as clinically relevant fungi including *Candida auris*. In this scenario, biotransformation has emerged as a complementary innovation strategy for antimicrobial discovery because it expands the chemical space around bioactive scaffolds through selective enzymatic or whole-cell modification. Among the available biocatalysts, fungi are especially attractive due to their metabolic plasticity and broad enzymatic repertoire, including cytochrome P450 monooxygenases, unspecific peroxygenases, laccases, peroxidases, and hydrolases. Current evidence shows that fungal systems can mediate regio- and stereoselective transformations of xenobiotics, aromatics, steroids, terpenes, and lipids, generating structurally refined metabolites of pharmacological and biotechnological interest. This narrative review discusses where fungal biotransformation currently stands as a platform for antimicrobial innovation, highlighting representative enzyme-characterized examples, the main fungal groups and catalytic systems involved, and the experimental workflows used to evaluate these processes. Particular emphasis is given to assay design with growing cells, resting cells, and isolated enzymes, as well as to analytical monitoring by time-course sampling, LC-HRMS/MS, dereplication, molecular networking, isolation, and structural elucidation. Overall, fungal biotransformation is presented as a discovery-enabling platform that links biodiversity, enzymatic catalysis, analytical chemistry, and biological prioritization in the search for new anti-infective molecules.

## 1 Introduction

Antimicrobial resistance (AMR) has become one of the major challenges of contemporary medicine, as it compromises the efficacy of essential therapies and increases mortality, length of hospital stay, and healthcare costs (Murray et al., 2022; World Health Organization, 2015, 2025c; O'Neill, 2016). This problem results both from the adaptive capacity of microorganisms and from intensified selective pressures driven by inappropriate antimicrobial use, failures in infection prevention and control, and inequalities in access to microbiological diagnosis (Murray et al., 2022; World Health Organization, 2015, 2025c; O'Neill, 2016). At the same time, the pace of introduction of new therapeutic agents remains below clinical needs, especially in light of the spread of resistance mechanisms among priority bacterial and fungal pathogens (Brown and Wright, 2016; Lewis, 2020; World Health Organization, 2025a,b).

Historically, natural products and their derivatives have played a central role in anti-infective drug discovery (Newman and Cragg, 2020; Atanasov et al., 2021). However, the incremental exploration of already known classes reaches its limits when widely disseminated resistance mechanisms begin to compromise the clinical performance of multiple antibiotics and antifungals, a process illustrated by the historical emergence of β-lactamases, MRSA, ESBLs, carbapenemases, vancomycin-resistant enterococci, and plasmid-mediated colistin resistance summarized in Table 1 (Brown and Wright, 2016; Lewis, 2020; World Health Organization, 2024). This mismatch has driven the search for innovation routes capable of expanding chemical space in an efficient and biologically informed manner, including computational and machine-learning approaches for structural prioritization and optimization (Newman and Cragg, 2020; Stokes et al., 2020; Atanasov et al., 2021).

**Table 1:** Historical milestones in antimicrobial resistance and global governance.

Year/Period	Milestone	Relevance to the antimicrobial crisis	References
1940	Description of a bacterial enzyme capable of destroying penicillin (β-lactamase)	Early demonstration that resistance may emerge even during the initial phase of therapeutic use	Abraham and Chain, 1940
1961	Report of methicillin-resistant *Staphylococcus aureus* (MRSA)	Landmark of Gram-positive resistance and rapid adaptation to penicillinase-resistant penicillins	Jevons, 1961
1980s−1990s	Dissemination and consolidation of ESBLs (extended-spectrum β-lactamases)	Expansion of resistance to third-generation cephalosporins, affecting empirical therapy and increasing carbapenem use	Bradford, 2001
1988	Report of vancomycin-resistant enterococci (VRE)	Reduction of therapeutic options for nosocomial Gram-positive infections and increased treatment complexity	Uttley et al., 1988
2001	Characterization of KPC-1 (carbapenemase)	Landmark for carbapenemases in Enterobacterales and the beginning of the threat to “last-line” drugs	Yigit et al., 2001
2009	Characterization of the *bla*_NDM − 1_ (metallo-β-lactamase) gene	Emergence of a mechanism with high dissemination potential and broad β-lactam hydrolysis spectrum	Yong et al., 2009
2010	Epidemiological evidence of NDM-1 expansion (India, Pakistan, and the UK)	Demonstration of transnational spread and the importance of surveillance/control linked to mobility	Kumarasamy et al., 2010
2016	Identification of the plasmid-mediated colistin resistance gene *mcr-1*	Threat to “last-resort” antibiotics and evidence of the role of mobile determinants across the human–animal–environment interface	Liu et al., 2016
2009 (and later global spread)	Initial description of *Candida auris*	Example of a fungal pathogen associated with hospital settings and therapeutic challenges due to resistance profiles	Satoh et al., 2009
2015	WHO Global Action Plan on AMR	Integrates action axes: awareness, surveillance, prevention/control, stewardship, and incentives for innovation	World Health Organization, 2015
2016	O'Neill Report (AMR Review)	Popularized estimates of economic impact and reinforced the need for new Research & Development incentive models	O'Neill, 2016
2022	WHO fungal priority pathogens list (FPPL)	Establishes global research and public health priorities for fungi, including antifungal resistance	World Health Organization, 2022
2024	WHO bacterial priority pathogens list (BPPL 2024)	Updates R&D and public health priorities against AMR, highlighting Gram-negative pathogens	World Health Organization, 2024
2025	WHO GLASS report with recent trends	Consolidates global analysis and reinforces the urgency of innovation, surveillance, and rational antimicrobial use	World Health Organization, 2025c

MRSA, methicillin-resistant Staphylococcus aureus; ESBLs, extended-spectrum β-lactamases; VRE, vancomycin-resistant enterococci; KPC, Klebsiella pneumoniae carbapenemase; NDM-1, New Delhi metallo-β-lactamase-1; mcr-1, mobilized colistin resistance 1; FPPL, fungal priority pathogens list; BPPL, bacterial priority pathogens list; GLASS, Global Antimicrobial Resistance and Use Surveillance System; WHO, World Health Organization.

In this context, biotransformation emerges as a particularly relevant technological strategy, because it enables the selective modification of organic substrates through cellular or enzymatic systems, often with high regio- and stereoselectivity (De Carvalho, 2017; Garzón-Posse et al., 2018; Bell et al., 2021). This approach is especially promising when applied to bioactive scaffolds of natural or semisynthetic origin, as it enables the generation of structural analogs, pharmacologically relevant metabolites, and variants with modulated physicochemical properties (Newman and Cragg, 2020; Atanasov et al., 2021; Esmaeili, 2025).

Among the various biocatalysts available, fungi occupy a prominent position due to their broad metabolic plasticity and the diversity of enzymes involved in oxidation, hydrolysis, and other structural transformations (Crešnar and Petrič, 2011; Hofrichter and Ullrich, 2014; Viswanath et al., 2014; Hofrichter et al., 2022). Rather than acting only as sources of new metabolites through biosynthesis, fungi can serve as versatile platforms for molecular modification, integrating intracellular xenobiotic metabolism and extracellular oxidative systems (Crešnar and Petrič, 2011; Khan et al., 2023; Monterrey et al., 2023; Melling et al., 2024).

Thus, this review is structured around four thematic axes: (i) the antimicrobial resistance crisis and the demand for new anti-infective agents; (ii) the conceptual and operational basis of biotransformation as a strategy for molecular diversification; (iii) the enzymatic and metabolic potential of fungi to generate structurally modified bioactive derivatives; and (iv) experimental and analytical workflows applicable to fungal biotransformation studies.

## 2 Methods

The literature search was conducted between August 2025 and March 2026 in the databases PubMed/MEDLINE, Scopus, Web of Science, and Google Scholar. The search covered primarily studies published from 2000 to 2026, while seminal articles published before 2000 were additionally incorporated when necessary to provide historical or conceptual background. Official documents from international health and surveillance agencies, particularly the World Health Organization (WHO), the Centers for Disease Control and Prevention (CDC), and the European Center for Disease Prevention and Control (ECDC), were also consulted to support the epidemiological and public-health framing of antimicrobial resistance (World Health Organization, 2015, 2022, 2025c; Centers for Disease Control Prevention, 2024; European Centre for Disease Prevention Control, 2024). This review was designed as a narrative review; therefore, no formal systematic review protocol, meta-analysis, or risk-of-bias assessment was applied.

Search strategies combined controlled terms and free-text keywords related to the central topics of the review, including: “antimicrobial resistance,” “antibiotic resistance,” “antifungal resistance,” “fungal biotransformation,” “fungal biocatalysis,” and “antimicrobial discovery.” Representative search combinations included expressions such as: (“antimicrobial resistance” OR “antifungal resistance”) AND (“fungal biotransformation” OR “fungal biocatalysis” OR “biocatalysis”); and (“fungi” OR “filamentous fungi”) AND (“antibacterial” OR “antifungal” OR “antimicrobial discovery”). Reference lists of selected articles and relevant reviews were also screened manually to identify additional studies of conceptual relevance.

## 3 The antimicrobial crisis and the search for new antimicrobials

Antimicrobial resistance should be understood as an evolutionary phenomenon accelerated by intense clinical, environmental, and agricultural selective pressure, with direct repercussions on the effectiveness of treating bacterial and fungal infections (Murray et al., 2022; World Health Organization, 2015, 2025c; O'Neill, 2016). Global estimates already demonstrate the significant impact of AMR on mortality and morbidity, while international reports reinforce the persistence of pathogen–antimicrobial combinations associated with high clinical burden (Murray et al., 2022; GBD 2021 Antimicrobial Resistance Collaborators, 2024; World Health Organization, 2025c). In particular, organisms such as Acinetobacter baumannii, Pseudomonas aeruginosa, Klebsiella pneumoniae, and Staphylococcus aureus continue to represent major public health concerns, alongside the expansion of clinically relevant fungal pathogens (World Health Organization, 2024, 2022; Satoh et al., 2009).

From a historical perspective, the trajectory of the antimicrobial crisis reveals a recurrent pattern: the introduction of new therapeutic options is followed, sooner or later, by the emergence and dissemination of resistance mechanisms. This process was already evident with the early description of bacterial enzymes capable of inactivating penicillin (Abraham and Chain, 1940), and continued over the decades with reports of MRSA (Jevons, 1961), the consolidation of extended-spectrum β-lactamases (Bradford, 2001), the emergence of vancomycin-resistant enterococci (Uttley et al., 1988), the expansion of carbapenemases such as KPC (Yigit et al., 2001) and NDM-1 (Yong et al., 2009; Kumarasamy et al., 2010), and the identification of plasmid-mediated colistin resistance via *mcr-1* (Liu et al., 2016). The temporal evolution of these milestones and their connection with global governance policies are summarized in Table 1.

This historical overview indicates that the problem lies not only in the emergence of new resistance mechanisms, but also in the difficulty of maintaining a sustained pace of therapeutic innovation (O'Neill, 2016; Brown and Wright, 2016; Lewis, 2020; World Health Organization, 2024). In recent decades, the discovery of new antibiotics and antifungals has faced economic, regulatory, and technical-biological barriers, including low relative commercial attractiveness, high translational failure rates, as well as permeability and efflux challenges, especially in Gram-negative bacilli (O'Neill, 2016; Brown and Wright, 2016; Lewis, 2020; World Health Organization, 2024). Recent WHO reports further emphasize that many compounds in development remain close to already known classes, which limits mechanistic novelty and robustness against resistance selection (World Health Organization, 2025a,b).

The antimicrobial crisis also extends beyond the bacterial axis. The emergence of Candida auris and the publication of the WHO fungal priority pathogens list demonstrate that antifungal resistance has become a strategic issue in global public health policies (World Health Organization, 2022; Satoh et al., 2009). Candida auris is a contemporary example of a fungal pathogen associated with healthcare settings, outbreak potential, difficult identification, and therapeutic challenges linked to resistance profiles. These examples reinforce the need for discovery platforms capable of generating both antibacterial and antifungal candidates through routes compatible with discovery, optimization, and functional evaluation in shorter cycles (World Health Organization, 2024, 2022, 2025b; Atanasov et al., 2021; Esmaeili, 2025).

Within this framework, biotransformation appears as a complementary strategy in the antimicrobial discovery ecosystem. By enabling the selective modification of bioactive scaffolds, it can contribute to the generation of analogs with improved activity, selectivity, and physicochemical properties, thereby connecting the traditional focus on the identification of bioactive natural products with contemporary approaches to structural prioritization and rational innovation (Newman and Cragg, 2020; Stokes et al., 2020; Atanasov et al., 2021; Esmaeili, 2025).

## 4 Biotransformation as a source of new antimicrobials

Biotransformation occupies a strategic position in antimicrobial innovation because it enables the selective expansion of the chemical space surrounding already known bioactive molecules (Newman and Cragg, 2020; Atanasov et al., 2021; Esmaeili, 2025). Rather than relying exclusively on total synthesis or multistep semisynthesis, this approach exploits the ability of whole cells or enzymes to introduce structural modifications such as hydroxylation, epoxidation, oxidation, reduction, hydrolysis, and oxidative cleavage, often associated with high selectivity (De Carvalho, 2017; Garzón-Posse et al., 2018; Bell et al., 2021).

This capability is particularly important in the anti-infective context because small structural changes may alter target interaction, susceptibility to efflux pumps, recognition by inactivating enzymes, and solubility and permeability properties (Brown and Wright, 2016; Lewis, 2020; Esmaeili, 2025). Thus, biotransformation serves as a tool for structural refinement, enabling the testing of structure–activity relationship hypotheses based on previously validated or promising scaffolds (Newman and Cragg, 2020; Atanasov et al., 2021; Esmaeili, 2025).

The contribution of biotransformation to antimicrobial innovation can also be interpreted mechanistically. Structural modification of known antimicrobial scaffolds may influence susceptibility to enzymatic inactivation, recognition by efflux systems, permeability across bacterial envelopes, and interaction with microbial targets (Brown and Wright, 2016; Lewis, 2020). Fungal biotransformation may therefore generate derivatives that preserve the bioactive core of a molecule while modifying functional groups related to stability, polarity, steric accessibility, or target binding. This is particularly relevant when resistance involves drug degradation, reduced intracellular accumulation, or impaired interaction with the antimicrobial target (World Health Organization, 2024, 2025c). Thus, biotransformation is not only a strategy for molecular diversification, but also a tool to explore how subtle structural changes may affect antimicrobial performance against resistant microorganisms.

Examples of this mechanistic logic include derivatives with reduced susceptibility to β-lactamase-mediated hydrolysis, especially when structural changes reduce access to labile pharmacophoric regions (Abraham and Chain, 1940; Bradford, 2001). In Gram-negative bacteria, modifications in polarity, charge distribution, hydrophobicity, or molecular size may affect outer membrane permeability and intracellular accumulation, two major barriers in antibacterial discovery (Brown and Wright, 2016; Lewis, 2020). Biotransformation may also support prodrug strategies by introducing or exposing functional groups that improve solubility, uptake, or intracellular activation. In addition, reactions such as hydroxylation, oxidation, reduction, glycosylation, methylation, or acylation may modify pharmacophores or peripheral substituents, generating analogs with altered activity, toxicity, or resistance profiles (De Carvalho, 2017; Garzón-Posse et al., 2018; Atanasov et al., 2021; Esmaeili, 2025).

From an operational standpoint, biotransformation may be conducted using whole cells, free enzymes, or biocatalytic cascades (De Carvalho, 2017; Garzón-Posse et al., 2018; Schrittwieser et al., 2018; Bell et al., 2021). Cellular systems are particularly valuable because they integrate multiple enzymes and continuously regenerate cofactors, whereas isolated enzymatic systems provide greater mechanistic clarity and selectivity control (De Carvalho, 2017; Garzón-Posse et al., 2018; Mordhorst and Andexer, 2020; Bell et al., 2021). The diversity of biocatalyst groups and their practical implications for antimicrobial discovery and optimization are summarized in Table 2.

**Table 2:** Biocatalyst groups and enzymatic classes relevant to biotransformation.

Biocatalyst group	Main enzymatic classes (examples)	Typical transformations	Main advantages/limitations	References
Bacteria (various)	Oxidoreductases, hydrolases, mono-/dioxygenases	Oxidations/reductions, selective hydrolyses, oxidative cleavages	High catalytic rate and accessible genetic engineering; often limited in highly selective oxyfunctionalization of complex molecules	De Carvalho, 2017; Garzón-Posse et al., 2018; Bornscheuer et al., 2012
Actinobacteria	P450s, transferases, monooxygenases	Modification of complex scaffolds, oxidations, conjugations	Strong track record in natural product metabolism; high biosynthetic diversity; may require culture and induction optimization	Sheldon and Woodley, 2018; Bornscheuer et al., 2012
Yeasts	Dehydrogenases/reductases; P450s (species/engineering dependent)	Enantioselective reductions, specific oxidations	Industrial robustness and useful expression chassis; substrate scope depends on strain and system design	De Carvalho, 2017; Faber, 2018
Filamentous fungi	Oxygenases (mono-/dioxygenases; cytochrome P450s), peroxidases (LiP/MnP/VP), phenoloxidases (laccases), unspecific peroxygenases (UPOs), hydrolases	Hydroxylations, epoxidations, aromatic oxidations, stereoselective biotransformations	High oxidative diversity and selectivity; good tolerance to complex substrates; growth-phase variation requires monitoring and proper controls	Crešnar and Petrič, 2011; Hofrichter and Ullrich, 2014; Hobisch et al., 2021; Molina-Espeja et al., 2014; Viswanath et al., 2014
Isolated enzymes (cell-free systems)	Oxygenases (P450s), unspecific peroxygenases, peroxidases, phenoloxidases (laccases), hydrolases, transferases	Hydroxylations, epoxidations, aromatic oxidations, stereoselective biotransformations	High reproducibility and clearer mechanistic attribution; may require cofactors/cofactor regeneration and stability optimization	Faber, 2018; Schrittwieser et al., 2018; Hofrichter and Ullrich, 2014; Hobisch et al., 2021; Molina-Espeja et al., 2014
Biocatalytic cascades	Multi-enzyme combinations (oxidases, reductases, transferases etc.)	Sequential one-pot or multi-step conversions	Rapid access to molecular complexity; integration requires compatibility between enzymes and reaction conditions	Schrittwieser et al., 2018

LiP, lignin peroxidase; MnP, manganese peroxidase; VP, versatile peroxidase; UPOs, unspecific peroxygenases; P450s, cytochrome P450 enzymes.

Among the microorganisms employed in biotransformation, fungi stand out due to their ability to metabolize natural products, xenobiotics, and structurally complex substrates (Crešnar and Petrič, 2011; Hofrichter and Ullrich, 2014; Hofrichter et al., 2022; Khan et al., 2023). This versatility derives from the combination of intracellular oxidative metabolism systems and extracellular systems capable of acting on aromatic, phenolic, and recalcitrant compounds (Crešnar and Petrič, 2011; Hobisch et al., 2021; Hofrichter et al., 2022). As a consequence, fungi can generate structurally refined analogs, pharmacologically relevant metabolites, and intermediates of interest for antimicrobial discovery (Khan et al., 2023; Atanasov et al., 2021; Esmaeili, 2025).

In addition to catalytic diversity, fungi offer important practical advantages, including relatively simple cultivation, good tolerance to hydrophobic substrates, and the possibility of up-scaling in flasks and bioreactors (Salter and Kell, 1995; García-Ochoa and Gomez, 2009; De Carvalho, 2017). When combined with modern metabolomics and mass spectrometry tools, fungal biotransformation becomes an even more powerful discovery platform, because the generation of chemical diversity is accompanied by robust strategies for detection, dereplication, and structural prioritization (Wang et al., 2016; Allard et al., 2016; Wolfender et al., 2019; Queiroz et al., 2024; Meunier et al., 2024). This combination of catalytic diversity, operational flexibility, and compatibility with advanced analytical workflows helps explain why fungi have become particularly attractive platforms for biotransformation-based strategies in antimicrobial discovery.

## 5 Fungal potential in biotransformation

The potential of fungi as biotransformation platforms derives from the functional breadth of their enzymatic repertoires, particularly cytochrome P450 systems, peroxygenases, laccases, peroxidases, and hydrolases (Crešnar and Petrič, 2011; Hofrichter and Ullrich, 2014; Viswanath et al., 2014; Hofrichter et al., 2022). These organisms combine intracellular xenobiotic metabolism systems with extracellular oxidative enzymes capable of acting on aromatic and hydrophobic substrates, creating a catalytic architecture suited to the selective modification of bioactive molecules (Crešnar and Petrič, 2011; Hobisch et al., 2021; Hofrichter et al., 2022).

Fungal biodiversity should not be interpreted as a homogeneous catalytic resource. Ascomycota include many filamentous fungi and yeasts widely explored in biotechnology, frequently associated with rapid growth, xenobiotic metabolism, and enzymes involved in oxidation, reduction, and hydrolysis (Crešnar and Petrič, 2011; Khan et al., 2023). In contrast, Basidiomycota are particularly relevant because of extracellular oxidative systems, including laccases, peroxidases, and unspecific peroxygenases, which participate in lignocellulose degradation and transformation of aromatic or recalcitrant substrates (Hofrichter and Ullrich, 2014; Viswanath et al., 2014; Hofrichter et al., 2022). Beyond these major lineages, endophytic, marine, extremophilic, mangrove-associated, and other underexplored environmental fungi may represent reservoirs of unusual catalytic activities. Fungi from competitive or extreme ecological niches may harbor stress-tolerance mechanisms and enzymatic systems capable of modifying complex chemical scaffolds, reinforcing biodiversity as a functional source of innovation for biotransformation-based antimicrobial discovery (Parshikov and Sutherland, 2015; Braga et al., 2024).

This taxonomic and ecological diversity also implies that part of the fungal metabolic potential may remain experimentally silent under conventional laboratory conditions.

Modern fungal biotransformation studies may also benefit from strategies designed to activate silent or poorly expressed metabolic pathways. Many fungi harbor biosynthetic and catabolic capacities that may remain inactive under standard laboratory conditions, and their expression may depend on culture conditions, co-cultivation, substrate induction, or stress exposure. When combined with metabolomics, genomics, transcriptomics, and proteomics, these strategies can help connect fungal taxonomy, ecological origin, enzymatic repertoire, and metabolite production (Wang et al., 2016; Allard et al., 2016; Wolfender et al., 2019). Multi-omics approaches may therefore improve fungal strain selection, clarify transformation pathways, and support the identification of metabolites with antimicrobial relevance (Khan et al., 2023; Queiroz et al., 2024; Meunier et al., 2024).

**Figure 1:**
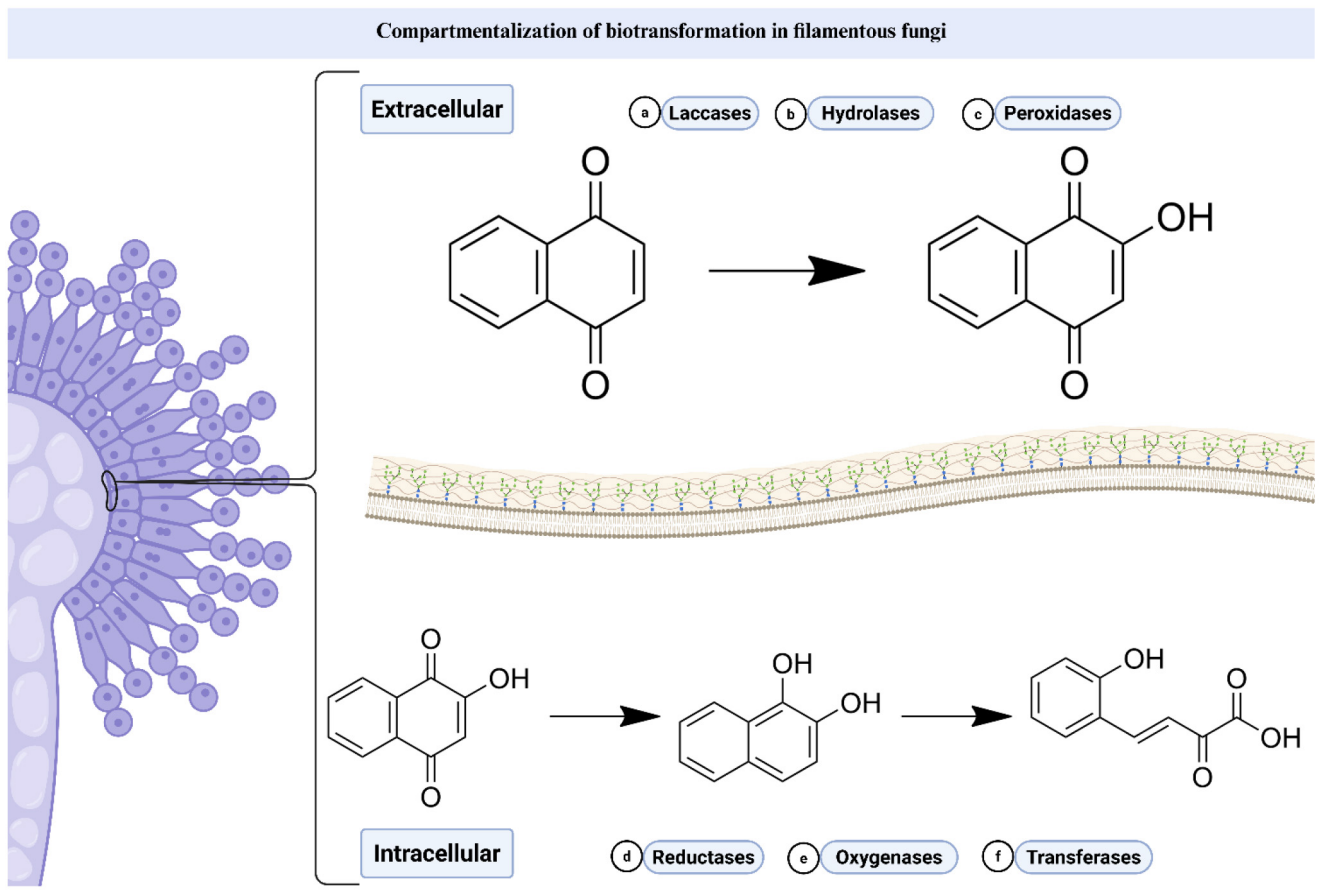
Compartmentalized fungal biotransformation. Schematic representation of a fungal hypha showing extracellular (a–c) and intracellular (d–f) systems involved in the conversion of an organic substrate into structurally modified metabolites. Oxidation and hydrolysis reactions predominate in the extracellular medium, whereas redox reactions, oxygenation reactions, and conjugation steps predominate intracellularly. (a) Laccases oxidize aromatic compounds using O_2_, generate radicals, and expand structural diversity. (b) Hydrolases cleave bonds by hydrolysis, releasing more polar and reactive products. (c) Peroxidases oxidize substrates in the presence of H_2_O_2_, forming reactive intermediates and oxidized derivatives. (d) Reductases reduce quinones and carbonyl groups using NAD(P)H, modulating reactivity and bioactivity. (e) Oxygenases insert oxygen atoms into the substrate structure. (f) Conjugation increases polarity and favors sequestration or efflux. The reaction scheme shown is a conceptual model illustrating how extracellular and intracellular enzymatic systems may act sequentially during fungal biotransformation. Based on the enzymatic systems and metabolic frameworks described for fungal xenobiotic metabolism, oxidative enzymes, and peroxygenase-mediated transformations (Crešnar and Petrič, 2011; Hofrichter and Ullrich, 2014; Khan et al., 2023; Monterrey et al., 2023). Created in BioRender. Souza, J. (2026) https://BioRender.com/9wp9ovf.

This arrangement is schematically represented in Figure 1, which highlights the compartmentalized nature of biotransformation in filamentous fungi, with extracellular and intracellular systems acting on the same substrate at different stages of transformation.

Among intracellular systems, cytochrome P450 enzymes deserve special attention for their ability to promote hydroxylation, epoxidation, demethylation, and other oxyfunctionalizations relevant to the metabolism of natural products and xenobiotics (Crešnar and Petrič, 2011). In applied terms, these enzymes enable the introduction of functional groups at positions that are difficult to access by conventional chemical synthesis, with high regioselectivity and often stereoselectivity (Faber, 2018; Bell et al., 2021; Crešnar and Petrič, 2011).

On the extracellular side, laccases, peroxidases, and related enzymes broaden the repertoire of possible transformations on aromatic, phenolic, and recalcitrant compounds (Viswanath et al., 2014; Hofrichter et al., 2022). These enzymes are relevant not only for biodegradation, but also for the generation of structurally modified derivatives of potential pharmacological interest (Viswanath et al., 2014; Hofrichter et al., 2022).

In parallel, recent reviews have reinforced the role of peroxygenases and related enzymes in peroxide-mediated oxygenation in fungal systems (Hofrichter and Ullrich, 2014; Hobisch et al., 2021; Molina-Espeja et al., 2014; Hofrichter et al., 2022; Monterrey et al., 2023; Poraj-Kobielska et al., 2011; De Gómez Santos et al., 2018). In fact, peroxygenases are among the most promising systems discussed in the literature. These enzymes are capable of performing selective oxyfunctionalizations using hydrogen peroxide as a cosubstrate (Hofrichter and Ullrich, 2014; Hobisch et al., 2021; Molina-Espeja et al., 2014; Hofrichter et al., 2022; Monterrey et al., 2023). Peroxygenases have been applied to the hydroxylation of aromatic compounds, the preparation of drug metabolites, terpene transformation, and the modulation between hydroxylation and epoxidation of lipid substrates (Kinne et al., 2009; Molina-Espeja et al., 2016; Carro et al., 2019; Monterrey et al., 2023; Melling et al., 2024).

**Figure 2:**
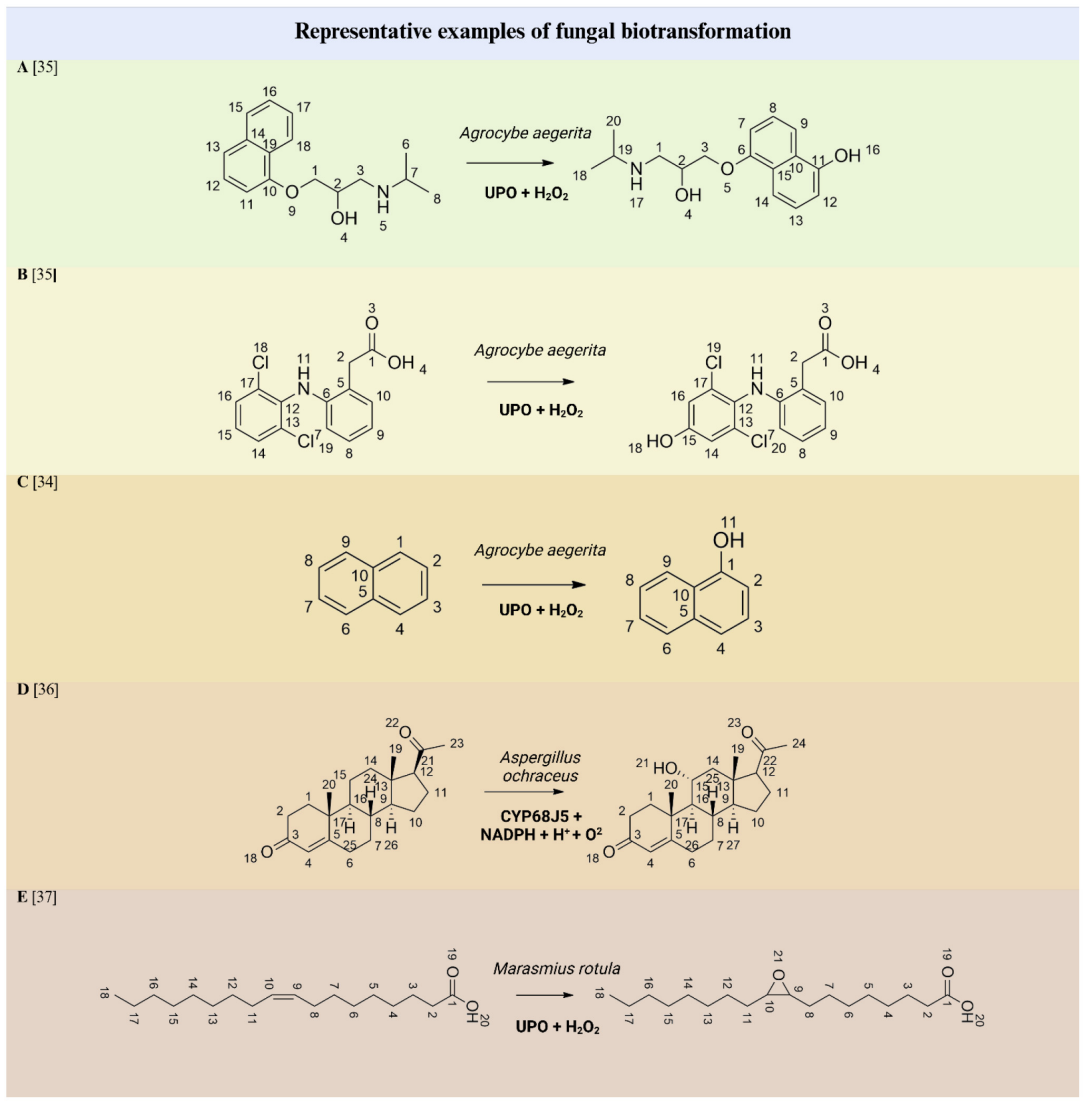
Representative enzyme-characterized fungal biotransformations. **(A)** Regioselective hydroxylation of propranolol catalyzed by the fungal unspecific peroxygenase (UPO) from *Agrocybe aegerita* in the presence of H_**2**_O_**2**_, yielding 5-hydroxypropranolol, a human-relevant drug metabolite that illustrates the applicability of fungal enzymes in selective pharmaceutical oxyfunctionalization. **(B)** Regioselective hydroxylation of diclofenac catalyzed by UPO from *A. aegerita* in the presence of H_**2**_O_**2**_, yielding 4′-hydroxydiclofenac, demonstrating the use of fungal peroxygenases for the preparation of relevant drug metabolites. **(C)** Hydroxylation of naphthalene catalyzed by UPO from *A. aegerita* in the presence of H_**2**_O_**2**_, producing 1-naphthol, highlighting the potential of fungal peroxygenases in selective aromatic oxyfunctionalization. **(D)** C11α steroid hydroxylation catalyzed by cytochrome P450 CYP68J5 from Aspergillus ochraceus (with NADPH as electron donor), yielding 11α-hydroxyprogesterone, exemplifying the role of fungal P450 systems in regioselective steroid derivatization. **(E)** Epoxidation of an unsaturated fatty acid catalyzed by UPO from *Marasmius rotula* in the presence of H_**2**_O_**2**_, yielding the corresponding epoxide, illustrating the catalytic versatility of fungal peroxygenases in lipid oxyfunctionalization. These examples demonstrate that fungal biotransformation can generate structurally diverse products with mechanistic, pharmacological, and biotechnological relevance. Sources: based on Kinne et al. (2009), Molina-Espeja et al. (2016), Qian et al. (2022), and Carro et al. (2019). Created in BioRender. Souza, J. (2026) https://BioRender.com/08y0l44.

Representative examples of fungal-mediated biotransformations with explicitly identified enzymes are shown in Figure 2. These cases were selected because they illustrate distinct but complementary biotechnological applications of fungal biocatalysis, including the selective generation of human-relevant drug metabolites, aromatic oxyfunctionalization, steroid derivatization, and lipid epoxidation. Together, they highlight how fungal peroxygenases and cytochrome P450 systems can be used not only to transform structurally diverse substrates, but also to generate products of pharmacological, mechanistic, and technological relevance.

The relevance of these enzymes for the discovery of new antimicrobials lies in their ability to generate derivatives that remain structurally close to the original scaffold while displaying altered physicochemical and pharmacological properties (Kinne et al., 2009; Molina-Espeja et al., 2016; Carro et al., 2019; Monterrey et al., 2023; Melling et al., 2024). Fungal systems have also been used for steroid and xenobiotic transformations of pharmaceutical interest (Qian et al., 2022; Wang et al., 2013; Mao et al., 2017; Al-Aboudi et al., 2008). In parallel, studies and reviews on terpenes, epoxide hydrolases, and environmentally relevant substrates reinforce the broader catalytic scope of fungi and fungal enzymes (Bicas et al., 2009; Bučko et al., 2023; De Souza Sevalho et al., 2023; Padilla-Garfias et al., 2024; Parshikov and Sutherland, 2015).

Representative examples of fungal biotransformation with explicitly identified enzymes, including fungal peroxygenases and cytochrome P450 systems, are summarized in Table 3 together with their main transformations and biotechnological relevance.

**Table 3:** Representative enzyme-characterized fungal biotransformations.

Substrate (before)	Fungus/enzyme (class)	Main transformation	Product (after)	Evidence of enzymatic attribution	Biotechnological relevance	References
Diclofenac	AaeUPO (unspecific peroxygenase from *Agrocybe aegerita*)	Regioselective aromatic hydroxylation	4′-Hydroxydiclofenac	*In vitro* assay with isolated fungal peroxygenase and product characterization	Demonstrates selective generation of a human-relevant drug metabolite and highlights fungal peroxygenases as tools for oxyfunctionalization of pharmaceutical compounds	Kinne et al., 2009
Propranolol	AaeUPO (unspecific peroxygenase from *Agrocybe aegerita*)	Regioselective hydroxylation	5-Hydroxypropranolol	*In vitro* assay with isolated fungal peroxygenase and product characterization	Illustrates the use of fungal enzymes for the selective preparation of pharmacologically relevant drug metabolites	Kinne et al., 2009
Naphthalene	UPO from *Agrocybe aegerita* (directed-evolution variant)	Aromatic hydroxylation	1-Naphthol	Enzyme engineering, directed evolution, and product characterization	Highlights the applicability of engineered fungal peroxygenases for selective aromatic oxyfunctionalization	Molina-Espeja et al., 2016
Progesterone	CYP68J5 (cytochrome P450 from *Aspergillus ochraceus*)	C11α steroid hydroxylation	11α-Hydroxyprogesterone	Identification of the P450, enzyme engineering, and validation in recombinant system	Demonstrates the relevance of fungal P450s for selective steroid derivatization and biotechnological steroid modification	Qian et al., 2022
Canrenone	CYP68J5 (cytochrome P450 from *Aspergillus ochraceus*)	C11α steroid hydroxylation	11α-Hydroxycanrenone	Attribution to the characterized fungal P450 system	Reinforces the potential of fungal P450s in regioselective functionalization of steroidal substrates of pharmaceutical interest	Qian et al., 2022
Unsaturated lipid model substrates	MroUPO (unspecific peroxygenase from *Marasmius rotula*)	Epoxidation vs. hydroxylation	Epoxides/alcohols	Mechanistic study with characterized fungal UPO	Shows the catalytic versatility of fungal peroxygenases and their applicability in selective lipid oxyfunctionalization	Carro et al., 2019

AaeUPO, unspecific peroxygenase from *Agrocybe aegerita*; MroUPO, unspecific peroxygenase from *Marasmius rotula*; UPO, unspecific peroxygenase; CYP, cytochrome P450 enzyme.

Although not exhaustive, the examples summarized in Figure 2 and Table 3 were selected because they represent well-characterized fungal biotransformations with explicit enzymatic attribution and clear pharmacological or biotechnological relevance. Taken together, Figures 1, 2, and Table 3 demonstrate that fungi are not only sources of new metabolites through biosynthesis, but also versatile platforms for the rational modification of chemical structures through biotransformation, thereby supporting their use in experimental workflows aimed at antimicrobial innovation.

## 6 Biotransformation experiments with fungi

Fungal biotransformation experiments should be understood as structured workflows designed to determine whether a given fungal system can convert a substrate of interest into chemically identifiable, experimentally reproducible, and biologically relevant products. In the context of antimicrobial discovery, the aim of these assays is not merely to demonstrate substrate conversion, but to evaluate whether fungal catalysis can expand the chemical and functional space around a bioactive scaffold in a way that justifies further pharmacological investigation. This distinction is important because the scientific value of fungal biotransformation lies not only in generating new metabolites, but in generating metabolites that can be analytically characterized, mechanistically interpreted, and biologically prioritized (Bell et al., 2021; Huang et al., 2024; Queiroz et al., 2024; Meunier et al., 2024).

**Figure 3:**
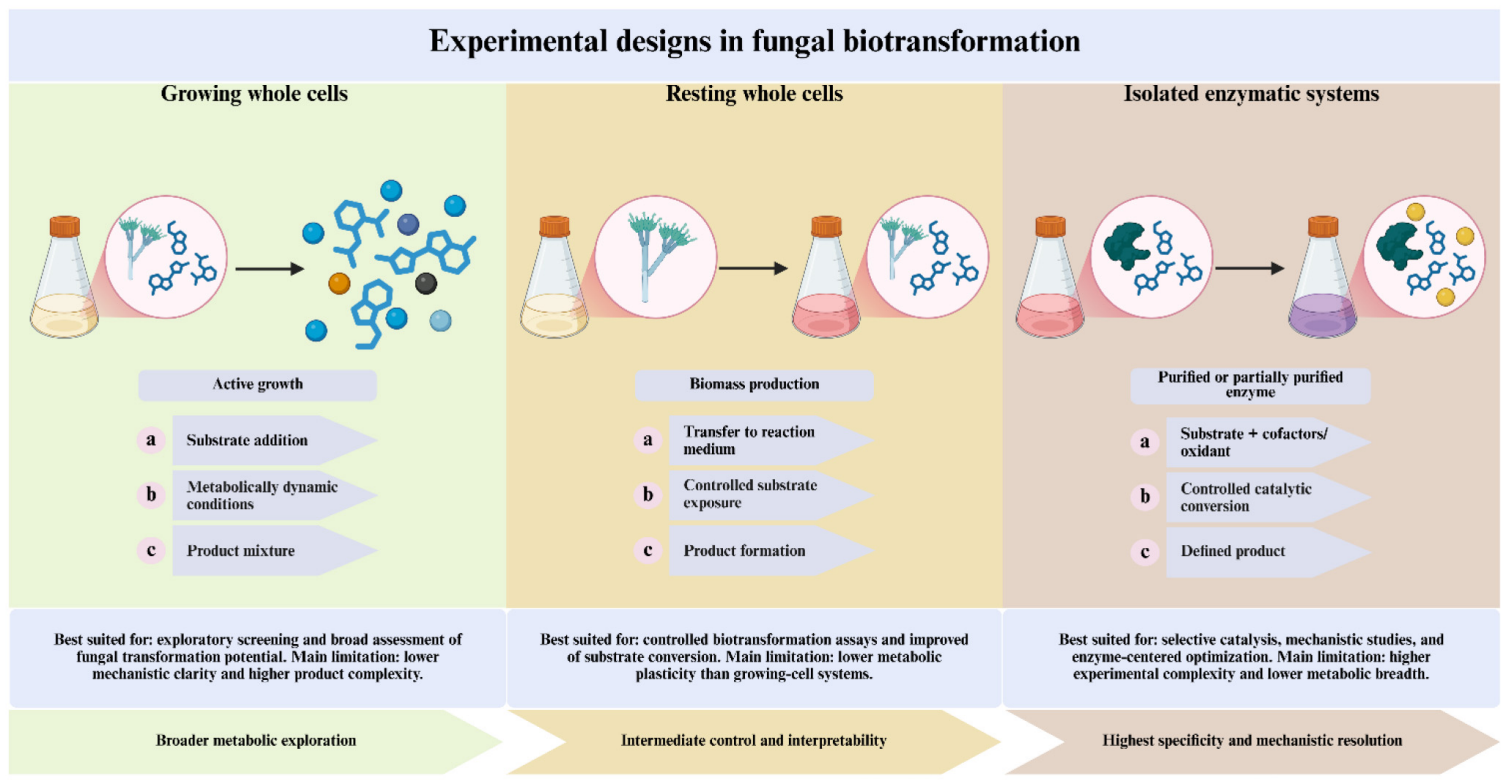
Main experimental designs in fungal biotransformation. Comparative schematic representation of the three main experimental approaches employed in fungal biotransformation assays: growing whole-cell systems, resting whole-cell systems, and isolated enzymatic systems. In growing whole-cell systems, the substrate is added during active fungal growth, allowing biotransformation under metabolically dynamic conditions and favoring broad exploratory screening, although often with greater product complexity. In resting whole-cell systems, previously produced fungal biomass is transferred to a defined reaction medium before substrate exposure, reducing interference associated with active growth and improving interpretability of substrate conversion. In isolated enzymatic systems, purified or partially purified enzymes are incubated with the substrate under controlled catalytic conditions, providing greater mechanistic clarity, selectivity, and suitability for enzyme-centered optimization. Together, these approaches represent a continuum from broader metabolic exploration to highly defined selective catalysis, illustrating how fungal biotransformation workflows can be tailored to different objectives in antimicrobial discovery. Based on the experimental frameworks discussed for whole-cell biocatalysis, isolated enzymatic systems, and process control (De Carvalho, 2017; Garzón-Posse et al., 2018; Bell et al., 2021; Mordhorst and Andexer, 2020). Created in BioRender. Souza, J. (2026) https://BioRender.com/08y0l44.

Once the organism and substrate have been selected, the experimental design must be defined according to the catalytic format to be used. As summarized in Figure 3, three main experimental approaches are commonly employed in fungal biotransformation studies: (a) growing whole-cell systems, (b) resting whole-cell systems, and (c) isolated enzymatic systems. These approaches do not represent equivalent technical alternatives, but rather different levels of metabolic complexity, catalytic control, and interpretative resolution.

In growing whole-cell systems, the substrate is added during active fungal growth, allowing the reaction to occur under metabolically dynamic conditions in which multiple enzymes may be induced or active simultaneously. This format is particularly useful for exploratory screening, because it reveals the global transformation potential of the organism and may uncover unexpected product profiles. However, this broader metabolic breadth often comes at the cost of greater product complexity and lower mechanistic clarity, since several parallel or sequential reactions may occur in the same culture system (De Carvalho, 2017; Garzón-Posse et al., 2018; Faber, 2018).

In resting whole-cell systems, fungal biomass is first produced under growth conditions and then transferred to a defined reaction medium before substrate exposure. This approach reduces interference associated with active growth, nutrient consumption, and ongoing biomass formation, thereby improving interpretability of substrate conversion and product formation. Resting-cell systems are especially useful when the aim is to obtain better experimental control without completely losing the catalytic advantages of whole-cell metabolism. They therefore occupy an intermediate position between exploratory growing-cell assays and more defined enzyme-based systems (De Carvalho, 2017; Garzón-Posse et al., 2018).

In isolated enzymatic systems, purified or partially purified fungal enzymes are incubated with the substrate under controlled catalytic conditions, usually in the presence of the required cofactors or oxidants. This format provides the highest mechanistic clarity and selectivity, since the reaction can be attributed to a defined catalytic entity rather than to the integrated metabolism of the whole organism. Such systems are especially valuable for enzyme-centered optimization, selective oxyfunctionalization, and preparative or mechanistic studies. Their main limitation is the higher experimental complexity, including enzyme preparation, stability control, cofactor regeneration, and oxidant management, as well as a lower overall metabolic breadth when compared with whole-cell systems (Sheldon and Woodley, 2018; Hofrichter and Ullrich, 2014; Bell et al., 2021; Mordhorst and Andexer, 2020).

The choice of fungal model depends on the objective of the study. Classical systems such as *Cunninghamella* spp. remain highly relevant in xenobiotic metabolism studies because of their recognized ability to mimic oxidative transformations and generate pharmacologically meaningful metabolites (Asha and Vidyavathi, 2009; Palmer-Brown et al., 2019). In contrast, environmental fungi, including isolates from tropical, mangrove-associated, or otherwise underexplored ecological niches, may provide broader or less predictable catalytic repertoires and are therefore especially valuable in exploratory studies aimed at revealing uncommon transformations or new metabolite patterns (Khan et al., 2023; Parshikov and Sutherland, 2015; Braga et al., 2024).

The substrate, in turn, should be selected according to a clear biological or chemical rationale, whether the goal is to obtain human-relevant drug metabolites, selectively modify natural products, or generate derivatives with altered physicochemical and pharmacological properties (Newman and Cragg, 2020; Atanasov et al., 2021; Esmaeili, 2025; Khan et al., 2023).

Analytical monitoring is the stage that transforms a fungal incubation into a meaningful discovery platform. Because fungal systems frequently generate multiple products simultaneously, time-course sampling is often necessary to monitor substrate depletion, detect primary and secondary metabolites, and follow conversion dynamics over time (De Carvalho, 2017; Garzón-Posse et al., 2018; Huang et al., 2024). At this point, liquid chromatography–high-resolution tandem mass spectrometry (LC-HRMS/MS), molecular networking, *in silico* fragmentation, dereplication workflows, and formula-filtering strategies become central tools for detecting, organizing, and prioritizing transformation products (Wang et al., 2016; Allard et al., 2016; Wolfender et al., 2019; Queiroz et al., 2024; Meunier et al., 2024). These tools are particularly important when the objective is not simply to confirm conversion, but to identify products that may warrant structural elucidation and biological testing.

In addition to LC-HRMS/MS-based metabolomics, fungal biotransformation workflows may be strengthened by genomics, transcriptomics, and proteomics. Genomic data can support the identification of enzyme families and gene clusters potentially involved in substrate transformation. Transcriptomic and proteomic analyses can indicate which pathways and enzymes are active under specific reaction conditions, especially when comparing control cultures, substrate-exposed cultures, and time-course samples. When integrated with metabolomics and molecular networking, these approaches may improve the interpretation of transformation pathways and increase the mechanistic resolution of fungal biotransformation studies (Wang et al., 2016; Allard et al., 2016; Wolfender et al., 2019; Queiroz et al., 2024; Meunier et al., 2024).

Once relevant metabolites have been detected, chromatographic isolation and structural characterization become necessary to validate their identity and support downstream pharmacological evaluation. At this stage, chromatographic separation, high-resolution mass spectrometry, and nuclear magnetic resonance (NMR) are the principal tools used to confirm product identity and purity (Wolfender et al., 2019; Queiroz et al., 2024; Kind and Fiehn, 2007). This is a decisive step for antimicrobial discovery, because a transformed metabolite only becomes a meaningful candidate when it can be isolated, structurally assigned, and submitted to reproducible biological evaluation.

The biological relevance of biotransformed metabolites must be demonstrated through integrated bioactivity evaluation. Analytical detection and structural elucidation are essential, but they are insufficient to support antimicrobial innovation without biological validation (Wolfender et al., 2019; Queiroz et al., 2024). Therefore, fungal biotransformation workflows should include antimicrobial screening followed by quantitative minimum inhibitory concentration (MIC) determination, evaluation of fungicidal or bactericidal activity when appropriate, cytotoxicity assays in mammalian cell models, and selectivity assessment. Anti-biofilm assays may also be important, since biofilm-associated tolerance is a major challenge in bacterial and fungal infections. Integrating chemical characterization with biological evaluation allows transformed metabolites to be prioritized by structural novelty, therapeutic relevance, and translational potential (Brown and Wright, 2016; Lewis, 2020; Newman and Cragg, 2020; Meunier et al., 2024; World Health Organization, 2025a).

When the objective is to obtain sufficient material for microbiological or pharmacological assays, scale-up introduces an additional layer of complexity. Transfer from exploratory incubations to larger flasks or bioreactors is not merely a volumetric adjustment, because oxygen transfer, agitation, fungal morphology, and reactor geometry may substantially alter both the yield and the product profile (García-Ochoa and Gomez, 2009). This is especially relevant in oxidative biotransformations, in which redox balance and oxygen availability may directly affect catalytic outcome. For this reason, process optimization should be viewed as part of the scientific rationale of fungal biotransformation and not merely as a technical afterthought (García-Ochoa and Gomez, 2009; Bell et al., 2021; Mordhorst and Andexer, 2020).

Taken together, fungal biotransformation experiments are most informative when they are organized as an integrated workflow: selection of a biologically meaningful substrate, definition of an appropriate fungal or enzymatic model, control of reaction conditions, time-resolved analytical monitoring, structural validation of the products obtained, and biological prioritization of the most relevant metabolites. From the perspective of antimicrobial innovation, the most useful assays are not simply those that show that a transformation occurred, but those that demonstrate how fungal catalysis can generate relevant, identifiable, and testable molecules with plausible pharmacological value (Bell et al., 2021; Huang et al., 2024; Queiroz et al., 2024; Meunier et al., 2024).

## 7 Integrative discussion

Antimicrobial resistance highlights the urgent need for new antibacterial and antifungal agents against priority pathogens, particularly multidrug-resistant Gram-negative bacteria, Staphylococcus aureus, and emerging resistant fungi such as Candida auris. Within this unmet therapeutic landscape, biotransformation should be viewed as a complementary discovery strategy capable of expanding the chemical and functional space around previously validated bioactive scaffolds (Brown and Wright, 2016; Lewis, 2020; World Health Organization, 2024; Garzón-Posse et al., 2018).

The value of biotransformation lies in its ability to generate useful chemical diversity without necessarily disrupting the prior bioactivity of the starting substrate (Bell et al., 2021; Esmaeili, 2025). In other words, biotransformation enables the exploration of biologically informed structural variation, which is especially valuable in compounds derived from natural products, semisynthetic compounds, or metabolites of pharmacological interest (Newman and Cragg, 2020; Atanasov et al., 2021; Esmaeili, 2025).

Fungi occupy a central role in this process because they combine intracellular oxidative metabolism systems with extracellular systems capable of acting on different chemical classes (Crešnar and Petrič, 2011; Hofrichter and Ullrich, 2014; Hofrichter et al., 2022; Khan et al., 2023). The combined action of P450 enzymes, laccases, peroxidases, unspecific peroxygenases, and hydrolases broadens the repertoire of possible transformations and supports the formation of metabolites that may be difficult to access through purely synthetic routes (Crešnar and Petrič, 2011; Hofrichter et al., 2022; Monterrey et al., 2023; Melling et al., 2024). Examples involving steroids, terpenes, aromatic compounds, and xenobiotics demonstrate that this potential is substantial and has already been experimentally demonstrated (Kinne et al., 2009; Molina-Espeja et al., 2016; Qian et al., 2022; Carro et al., 2019; Melling et al., 2024). In addition, studies on the degradation and transformation of polycyclic aromatic hydrocarbons by fungi, including yeasts, show that the fungal repertoire also extends to recalcitrant substrates of chemical and biotechnological interest (Padilla-Garfias et al., 2024).

Importantly, the literature already provides representative proof-of-concept cases involving the transformation of drugs, aromatic compounds, steroids, and lipids, showing that fungal biotransformation has progressed beyond theoretical promise and now offers experimentally demonstrated routes to structurally diversified metabolites. However, generating chemical diversity does not automatically translate into pharmaceutical innovation. The transition from a transformed metabolite to a relevant antimicrobial candidate depends on additional filters, including reproducible biological activity, adequate selectivity, production feasibility, chemical stability, scalability, and clear pharmacological rationale (Brown and Wright, 2016; Lewis, 2020; World Health Organization, 2025c; Atanasov et al., 2021; Esmaeili, 2025). Many biotransformation systems are excellent for proof of concept, but less efficient when simplicity of operation, high yield, or selective production of a single metabolite is required (Sheldon and Woodley, 2018; García-Ochoa and Gomez, 2009; Huang et al., 2024).

For this reason, integration with modern analytical platforms is decisive. In fungal systems, the simultaneous formation of multiple products is frequent, making liquid chromatography–high-resolution tandem mass spectrometry (LC-HRMS/MS), molecular networking, dereplication, preparative chromatography, and structural prioritization strategies indispensable (Wang et al., 2016; Allard et al., 2016; Wolfender et al., 2019; Queiroz et al., 2024; Meunier et al., 2024). Without such infrastructure, the diversity generated by biotransformation remains underexplored. In parallel, recent methodological reviews on biocatalysis and cofactor regeneration strategies reinforce that the translational maturity of these processes depends as much on catalytic performance as on process control (Bell et al., 2021; Mordhorst and Andexer, 2020).

Another key point is that the translational value of biotransformation increases when it is linked to microbial biodiversity prospecting. Fungi from poorly explored niches, including tropical and mangrove-associated environments, may harbor unusual catalytic repertoires and reveal less predictable transformation routes (Khan et al., 2023; Parshikov and Sutherland, 2015; Braga et al., 2024). In this sense, fungal biotransformation serves as an interface technology connecting biodiversity, medicinal chemistry, microbiology, and enzymatic biochemistry (Khan et al., 2023; Monterrey et al., 2023; Melling et al., 2024; Meunier et al., 2024; Bell et al., 2021).

In conclusion, the present review indicates that fungal biotransformation has moved from a largely descriptive phenomenon to a strategically relevant platform for antimicrobial innovation. Its value lies not only in the ability to generate new metabolites, but in the selective generation of chemically identifiable, mechanistically interpretable, and biologically testable derivatives from pharmacologically meaningful scaffolds. Current evidence supports the relevance of fungal systems ranging from classical xenobiotic-transforming fungi to enzyme-centered platforms based on cytochrome P450s and unspecific peroxygenases, complemented by laccases, peroxidases, and hydrolases. At the same time, the field still faces an important translational challenge: to connect catalytic diversification with rigorous antimicrobial evaluation, selectivity testing, structural elucidation, and scalable production. Therefore, the future impact of fungal biotransformation will depend on how effectively biodiversity prospecting, biocatalysis, high-resolution analytical monitoring, and biological prioritization are integrated to deliver new antibacterial and antifungal candidates. In this sense, fungal biotransformation should be considered not only a chemical diversification tool, but also an integrative microbiological strategy for transforming fungal biodiversity into testable anti-infective innovation.
